# Paeoniflorin Promotes Ovarian Development in Mice by Activating Mitophagy and Preventing Oxidative Stress

**DOI:** 10.3390/ijms25158355

**Published:** 2024-07-30

**Authors:** Huaming Xi, Ziqian Wang, Minghui Li, Xing Duan, Yuan Li

**Affiliations:** Key Laboratory of Applied Technology on Green-Eco-Healthy Animal Husbandry of Zhejiang Province, Zhejiang Provincial Engineering Laboratory for Animal Health Inspection & Internet Technology, Zhejiang International Science and Technology Cooperation Base for Veterinary Medicine and Health Management, China-Australia Joint Laboratory for Animal Health Big Data Analytics, College of Animal Science and Technology & College of Veterinary Medicine, Zhejiang A&F University, Hangzhou 311300, China; xihuaming@zafu.edu.cn (H.X.);

**Keywords:** Paeoniflorin, ovary, autophagy, oxidative stress, follicle

## Abstract

During the development of animal organs, various adverse stimuli or toxic environments can induce oxidative stress and delay ovarian development. Paeoniflorin (PF), the main active ingredient of the traditional Chinese herb *Paeonia lactiflora* Pall., has protective effects on various diseases by preventing oxidative stress. However, the mechanism by which PF attenuates oxidative damage in mouse ovaries remains unclear. We evaluated the protective effects of PF on ovaries in an H_2_O_2_-induced mouse oxidative stress model. The H_2_O_2_-induced mouse ovarian oxidative stress model was used to explore the protective effect of PF on ovarian development. Histology and follicular development were observed. We then detected related indicators of cell apoptosis, oxidative stress, and autophagy in mouse ovaries. We found that PF inhibited H_2_O_2_-induced ovarian cell apoptosis and ferroptosis and promoted granulosa cell proliferation. PF prevented oxidative stress by increasing nuclear factor erythroid 2-related factor 2 (Nrf2) and heme oxygenase-1 (HO-1) expression levels. In addition, the autophagic flux of ovarian cells was activated and was accompanied by increased lysosomal biogenesis. Moreover, PF-mediated autophagy was involved in clearing mitochondria damaged by H_2_O_2_. Importantly, PF administration significantly increased the number of primordial follicles, primary follicles, secondary follicles, and antral follicles. PF administration improved ovarian sizes compared with the H_2_O_2_ group. The present study suggested that PF administration reversed H_2_O_2_-induced ovarian developmental delay and promoted follicle development. PF-activated mitophagy is crucial for preventing oxidative stress and improving mitochondrial quality.

## 1. Introduction

The ovaries play a key role in maintaining female reproductive capacity. Exposure to toxic environments or stress stimuli can cause ovarian oxidative stress. Oxidative stress is a state of intracellular oxidation and antioxidant imbalance and is considered to be an important factor in damaging reproductive performance [1,2]. The main source of reactive oxygen species (ROS) in cells is mitochondria [3]. Mitochondrial dysfunction is generally considered to be responsible for the excessive accumulation of ROS. The excessive accumulation of ROS further damages the mitochondria and ultimately accelerates the apoptosis process. Oxidative stress causes the excessive production of ROS and damages cell mitochondrial function. An increasing number of studies have shown that the ovaries are susceptible to oxidative stress during development [4,5,6]. Many studies have confirmed that oxidative stress contributes to female infertility [1,7,8]. Oxidative stress in the ovary can delay follicular development, cause follicular atresia, and lead to female infertility [8]. Shen et al. [9] suggested that oxidative stress impairs granulosa cell function in mice and induces apoptosis. However, the detailed mechanisms by which oxidative stress induces arrested ovarian development and follicular atresia remain largely unknown.

Autophagy is an important mechanism for cells to maintain homeostasis and can participate in the removal of damaged organelles and proteins. To maintain mitochondrial quality, cells are able to selectively remove damaged mitochondria by mitophagy. Normal mitochondrial activity is crucial to cell function, and the timely elimination of damaged mitochondria is a self-protection mechanism in cells. Mitophagy prevents damaged mitochondria from further damaging cells. PTEN-induced kinase 1 (PINK1) interacts with Parkin to jointly regulate the mitophagy process to maintain mitochondrial quality. Jiang et al. found that melatonin inhibits the PINK1–Parkin pathway and mitophagy to protect mouse granulosa cells from oxidative damage [10]. Adverse stimuli such as starvation, organelle damage, and oxidative stress can induce an increase in autophagy activity. Oxidative stress can cause excessive production of ROS and induce autophagy in granulosa cells [11]. Furthermore, Zhang et al. [12] reported that oxidative stress induces follicular atresia and reduces oocyte developmental capacity in mice. Autophagy of germ cells in mouse ovaries is critical to maintaining the primordial follicle pool [13,14]. Gioia et al. [15] suggested that autophagy inhibits apoptosis in porcine granulosa cells. These results indicate that autophagy plays a dual role in oxidative damage and ovarian development. However, the regulatory role of autophagy in H_2_O_2_-induced ovarian oxidative stress still needs further study.

Paeoniflorin (PF) is the principal bioactive constituent isolated from the traditional Chinese herb (*Paeonia lactiflora* Pall.). PF has anti-inflammatory, immunomodulatory, and antioxidative effects [16,17,18]. Research has demonstrated that PF inhibits oxidative stress and autophagy through the Nrf2/HO-1 signaling pathway in human umbilical vein endothelial cells [19]. In addition, Wang et al. found that PF activates the Akt/Nrf2/GPX4 pathway to prevent ferroptosis [20]. In the rat polycystic ovarian syndrome model, PF treatment decreased TGF-β1 and Smad3 expression levels and delayed ovarian fibrosis [21]. Wu et al. [22] reported that PF administration improves the ovarian index and follicle development in diminished ovarian reserve mice and promotes the synthesis of estradiol in ovarian granulosa-like KGN cells. *Paeonia lactiflora* Pall., a traditional Chinese medicine, is mostly used to improve female fertility and ovarian function [23]. Paeoniflorin is the main active ingredient of *Paeonia lactiflora* Pall., but its regulatory role and molecular mechanism in ovarian oxidative stress have not been determined.

In this study, the H_2_O_2_-induced mouse ovarian oxidative stress model was used to explore the protective effect of PF on ovarian development under H_2_O_2_-induced oxidative damage and to clarify its underlying mechanism. We found that PF effectively improved the development of ovaries and follicles in H_2_O_2_-induced oxidative damage in mouse ovaries by activating autophagy, inhibiting oxidative stress, and reducing cell apoptosis.

## 2. Results

### 2.1. H_2_O_2_-Induced Ovarian Developmental Delay in Mice Is Attenuated by PF Administration

**Figure 1:**
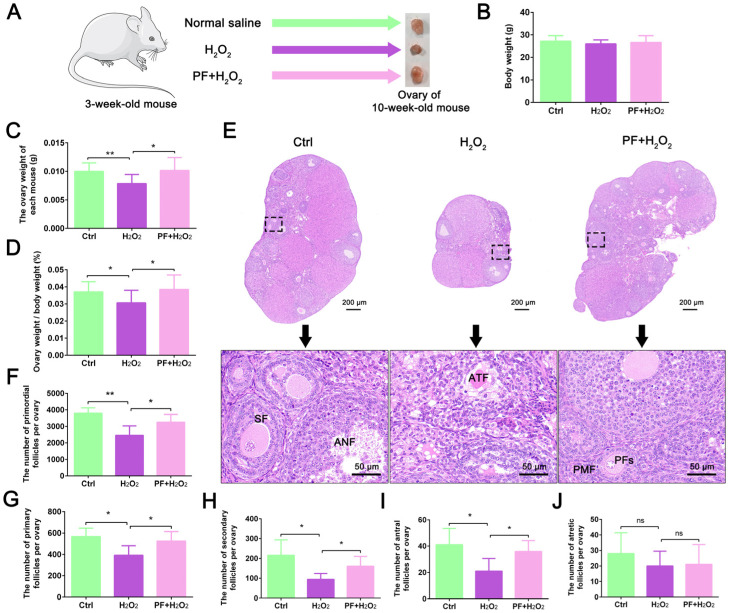
**Effects of PF on H_2_O_2_-induced ovarian developmental delay in mice**. (**A**) Mice were treated with normal saline, H_2_O_2_, or PF. (**B**) Quantification analysis of body weight (n = 10). (**C**) Quantification analysis of ovary weight (n = 10). (**D**) Quantification analysis of ovary weight/body weight (n = 10). (**E**) Histological changes in ovaries were examined by hematoxylin and eosin staining. PMF, primordial follicle. PFs, primary follicles. SF, secondary follicle. ANF, antral follicle. ATF, atretic follicle. (**F**–**J**) Quantification of the number of primordial follicles, primary follicles, secondary follicles, antral follicles, and atretic follicles (n = 5). Data represent mean ± SEM. Ctrl, control group. PF + H_2_O_2_, Paeoniflorin + H_2_O_2_ group. ns, no significance. * *p* < 0.05, ** *p* < 0.01.

To determine the protective effect of PF on ovarian development, we established an oxidative stress model targeting mouse ovaries using H_2_O_2_. After H_2_O_2_ and PF treatment of 3-week-old mice for 7 weeks, ovarian tissues from each group were collected (Figure 1A). The results showed that the body weight of mice did not change significantly in the H_2_O_2_ and PF + H_2_O_2_ groups (*p* > 0.05) compared with the control group (Figure 1B). After H_2_O_2_ treatment, the ovary weight and ovary weight/body weight significantly decreased (*p* < 0.05), while PF administration reversed the damage due to H_2_O_2_ treatment (*p* < 0.05, Figure 1C,D). Further results revealed that H_2_O_2_-treated mice displayed more impaired ovarian follicle structures than mice in the control and PF + H_2_O_2_ groups. PF treatment promoted follicle development and improved ovarian sizes compared with the H_2_O_2_ group (Figure 1E). Moreover, we counted the number of primordial follicles, primary follicles, secondary follicles, antral follicles, and atretic follicles in histological sections of ovaries for each group. H_2_O_2_-induced oxidative damage resulted in a significant reduction (*p* < 0.05) in the number of primordial follicles, primary follicles, secondary follicles, and antral follicles (Figure 1F–I). In contrast, PF administration significantly increased (*p* < 0.05) the number of follicles of various types and improved follicle development (Figure 1F–I). However, no significant changes were found (*p* > 0.05) in the number of atretic follicles in each treatment group (Figure 1J). The results suggested that PF administration reversed H_2_O_2_-induced ovarian developmental delay in mice.

### 2.2. PF Inhibits H_2_O_2_-Induced Cell Apoptosis and Promotes Granulosa Cell Proliferation

**Figure 2:**
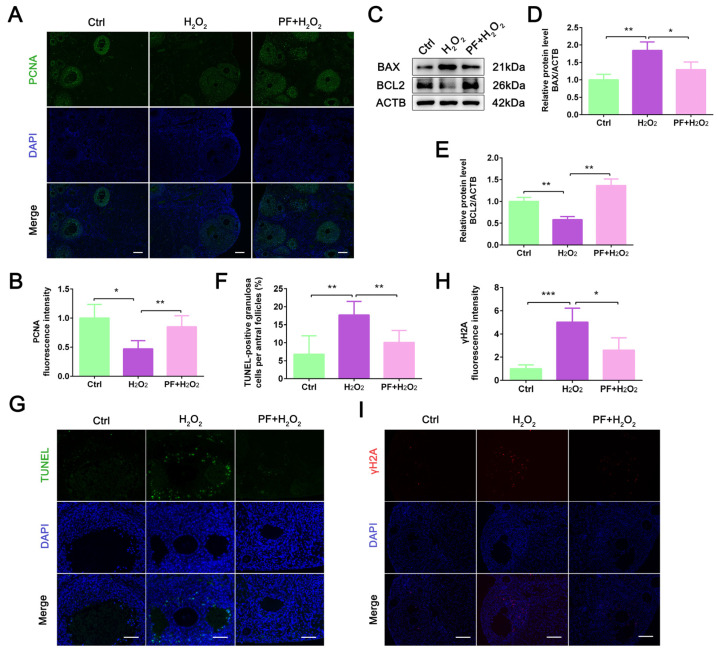
**PF promotes granulosa cell proliferation and inhibits H_2_O_2_-induced ovarian cell apoptosis**. (**A**,**B**) Representative immunofluorescence images and analysis of PCNA in ovary sections (Bar = 100 μm). (**C**–**E**) Western blot and quantification of BAX and BCL2 in ovarian tissues (n = 6). ACTB served as an internal control. (**F**,**G**) Quantification of TUNEL-positive cell numbers (Bar = 50 μm). (**H**,**I**) Representative immunofluorescence images and analysis of γH2A in ovary sections (Bar = 50 μm). Data represent mean ± SEM. Ctrl, control group. PF + H_2_O_2_, Paeoniflorin + H_2_O_2_ group. * *p* < 0.05, ** *p* < 0.01, *** *p* < 0.001.

An increase in the number of apoptotic cells can lead to a reduction in organ size. To explore whether the reduction in ovarian size caused by H_2_O_2_ is related to ovarian cell apoptosis, we analyzed ovarian cell proliferation and apoptosis. IF staining results showed that the PCNA-positive signal was specifically localized in granulosa cells (Figure 2A). H_2_O_2_ treatment significantly decreased (*p* < 0.05) the PCNA fluorescence intensity in ovaries, while PF administration significantly increased (*p* < 0.01) the expression level of PCNA (Figure 2B). Moreover, Western blot results showed that PF administration decreased the expression level of BAX (*p* < 0.05) and increased the BCL2 expression level (*p* < 0.01) in the ovaries (Figure 2C–E). PF administration consistently, significantly reduced (*p* < 0.01) the number of TUNEL-positive granulosa cells in the PF + H_2_O_2_ group compared with the H_2_O_2_ group (Figure 2F,G). PF administration significantly decreased (*p* < 0.05) the γH2A fluorescence intensity in the PF + H_2_O_2_ group (Figure 2H,I), indicating that H_2_O_2_-induced DNA damage was rescued by PF administration. The results suggested that PF treatment inhibited cell apoptosis and promoted granulosa cell proliferation.

### 2.3. PF Prevents Oxidative Stress and Inhibits Ferroptosis in Mouse Ovaries

**Figure 3:**
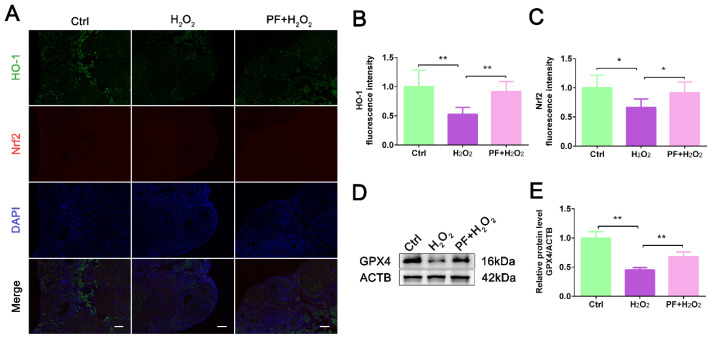
**PF prevents oxidative stress and inhibits ferroptosis in mouse ovaries**. (**A**–**C**) Representative immunofluorescence images and analysis of HO-1 and Nrf2 in ovary sections (Bar = 100 μm). (**D**,**E**) Western blot and quantification of GPX4 in ovarian tissues (n = 6). ACTB served as an internal control. Data represent mean ± SEM. Ctrl, control group. PF + H_2_O_2_, Paeoniflorin + H_2_O_2_ group. * *p* < 0.05, ** *p* < 0.01.

To determine whether PF prevented H_2_O_2_-induced oxidative stress to promote cell survival, we analyzed the expression of Nrf2 and HO-1 in mouse ovaries. IF staining showed that in the PF + H_2_O_2_ group, PF administration significantly increased (*p* < 0.05) Nrf2 and HO-1 expression levels compared with the H_2_O_2_ group (Figure 3A–C). The results of Western blot showed that H_2_O_2_ treatment decreased the GPX4 expression level (*p* < 0.01) compared with the control group, and PF administration caused a significant increase (*p* < 0.01) in the GPX4 expression level in the PF + H_2_O_2_ group (Figure 3D,E), indicating that PF improved the ability of ovarian cells to resist ferroptosis. The results suggested that H_2_O_2_-induced oxidative stress and ferroptosis in ovarian cells were inhibited by PF.

### 2.4. PF Activates Autophagy Activity and Lysosomal Biogenesis

**Figure 4:**
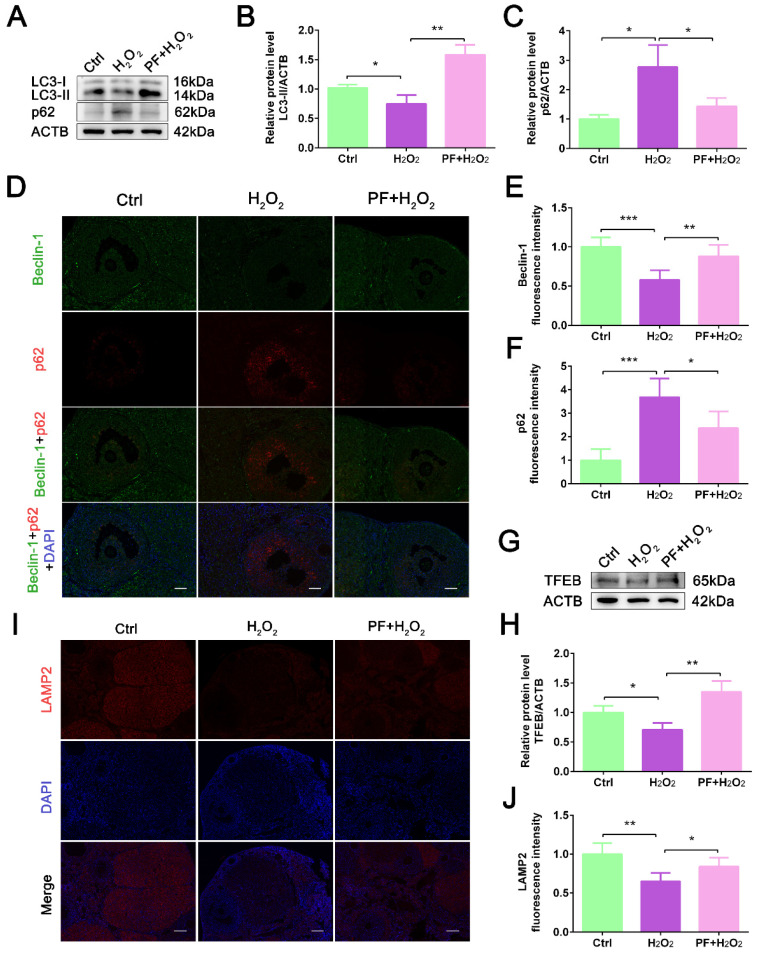
**PF activates autophagy activity and lysosomal biogenesis in the mouse ovaries**. (**A**–**C**) Western blot and quantification of LC3-II and p62 in ovarian tissues (n = 6). ACTB served as an internal control. (**D**–**F**) Representative immunofluorescence images and analysis of Beclin-1 and p62 in ovary sections (Bar = 100 μm). (**G**,**H**) Western blot and quantification of TFEB in ovarian tissues (n = 6). ACTB served as an internal control. (**I**,**J**) Representative immunofluorescence images and analysis of LAMP2 in ovary sections (Bar = 100 μm). Data represent mean ± SEM. Ctrl, control group. PF + H_2_O_2_, Paeoniflorin + H_2_O_2_ group. * *p* < 0.05, ** *p* < 0.01, *** *p* < 0.001.

Autophagy plays a very important role in oxidative stress. To explore whether autophagy is involved in the protective effect of PF against ovarian oxidative damage, we detected the expression levels of autophagy-related proteins. The results showed that H_2_O_2_ significantly decreased (*p* < 0.05) the LC3-II expression level and promoted p62 protein accumulation (*p* < 0.05, Figure 4A–C), indicating that H_2_O_2_-induced oxidative stress reduced autophagy activity in ovarian cells. PF treatment significantly upregulated LC3-II expression level (*p* < 0.01), leading to a decrease in the p62 protein level (Figure 4A–C). IF staining consistently showed that PF treatment increased the Beclin-1 fluorescence intensity (*p* < 0.01) and decreased p62 fluorescence intensity (*p* < 0.05) compared with the H_2_O_2_ group (Figure 4D–F). Furthermore, the TFEB expression level was significantly upregulated (*p* < 0.01) in ovaries after PF treatment (Figure 4G,H). PF administration increased (*p* < 0.05) LAMP2 expression level in the PF+ H_2_O_2_ group (Figure 4I,J), suggesting that PF administration activated autophagy activity and lysosomal biogenesis in ovarian cells.

### 2.5. PF Promotes Mitophagy to Improve Mitochondrial Quality

**Figure 5:**
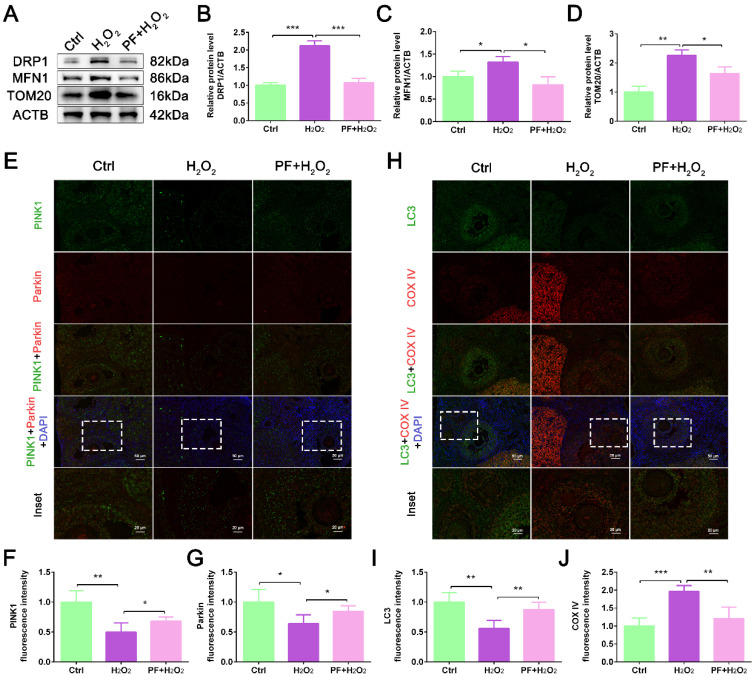
**PF promotes mitophagy to improve mitochondrial quality**. (**A**–**D**) Western blot and quantification of DRP1, MFN1, and TOM20 in ovarian tissues (n = 6). ACTB served as an internal control. (**E**–**G**) Representative immunofluorescence images and analysis of PINK1 and Parkin in ovary sections (Bar = 100 μm). (**H**–**J**) Representative immunofluorescence images and analysis of LC3 and COX IV in ovary sections (Bar = 100 μm). Data represent mean ± SEM. Ctrl, control group. PF + H_2_O_2_, Paeoniflorin + H_2_O_2_ group. * *p* < 0.05, ** *p* < 0.01, *** *p* < 0.001.

Oxidative stress can cause mitochondrial dysfunction, leading to cell apoptosis. To determine whether mitochondrial function is affected by H_2_O_2_ and PF, mitochondria-related protein expression levels were detected. Western blot results showed that H_2_O_2_ treatment significantly increased (*p* < 0.05) the expression levels of fusion protein MFN1, fission protein DRP1, and mitochondrial membrane protein TOM20. At the same time, PF administration significantly decreased (*p* < 0.05) the expression levels of these proteins (Figure 5A–D). Mitophagy can clear damaged mitochondria to maintain mitochondrial quality. We further analyzed mitophagy levels in different treatment groups. IF staining showed that PF administration increased the fluorescence intensities of PINK1 and Parkin in the PF + H_2_O_2_ group compared with the H_2_O_2_ group (*p* < 0.05, Figure 5E–G). Importantly, PF administration markedly increased the co-localization of LC3 and COX IV (Figure 5H–J), indicating that PF promoted mitophagy to maintain mitochondrial quality in oxidative damage to the ovary induced by H_2_O_2_.

## 3. Discussion

Oxidative stress can delay follicular development and reduce ovarian function, leading to reduced female reproductive capacity and even infertility. More and more adverse factors or toxic substances cause oxidative stress and affect ovarian function. PF, as the main active ingredient of the traditional Chinese herb *Paeonia lactiflora* Pall., can improve ovarian function. In this study, we found that (1) PF protected ovarian cells from H_2_O_2_-induced oxidative damage and inhibited ferroptosis; (2) PF reduced the amount of ovarian cell apoptosis and promoted ovarian development; (3) PF activated mitophagy to maintain mitochondrial quality control in ovaries.

Oxidative stress is a phenomenon that is an imbalance between the production of ROS and the antioxidant defenses of cells. Many studies have demonstrated that female infertility is related to oxidative stress [1,24,25]. The H_2_O_2_-induced oxidative stress model is widely used to explore the molecular mechanisms related to oxidative damage. The present study found that H_2_O_2_ caused oxidative stress, while PF treatment increased the expression levels of Nrf2 and HO-1, which is consistent with previous study [16]. Nrf2 is a crucial regulator of cellular antioxidant capacity, but its overactivation also favors cancer progression. Previous studies have suggested that increasing the Nrf2 expression level can improve ovarian antioxidant capacity and promote ovarian development [26,27]. Ren et al. [16] reported that PF activates the Nrf2/HO-1 signaling pathway to improve antioxidant capacity in H9c2 cells. The pterostilbene-activated Nrf2/HO-1 signaling pathway protected human ovarian granulosa cells from oxidative stress and ferroptosis [28]. Spermidine consistently inhibits oxidative stress and ferroptosis and alleviates ovarian damage by the Nrf2/HO-1/GPX4 pathway [26]. These studies suggest that Nrf2 plays a critical role in ovarian resistance to oxidative damage. In addition, GPX4 is a key enzyme that clears lipid peroxides, and its expression level is regulated by the transcription factor Nrf2 [29]. Ma et al. [30] confirmed that PF inhibits ferroptosis in a mouse acute kidney injury model and human renal tubular epithelial cells. We observed that PF elevated the GPX4 protein level in ovarian tissues, consistent with the results of a previous study [30], which indicated that PF protected the ovary against oxidative stress by activating the Nrf2/HO-1 signaling pathway.

In this study, H_2_O_2_-induced oxidative stress delayed ovarian development and reduced ovarian size. Previous study has suggested that reduced organ size is associated with increased numbers of apoptotic cells [31]. Apoptosis plays an important role in ovarian function and follicle development [32]. BAX is a pro-apoptotic protein, while BCL2 is an anti-apoptotic protein. We found that H_2_O_2_ treatment upregulated BAX expression levels and downregulated BCL2 expression levels, which indicated that H_2_O_2_ caused excessive apoptosis of ovarian cells. Therefore, oxidative stress caused by H_2_O_2_ delays ovarian development possibly by increasing ovarian cell apoptosis, and apoptosis caused by H_2_O_2_ is the direct cause of the reduction in ovarian size. Under oxidative stress conditions, PF prevented oxidative damage and reduced the number of ovarian apoptotic cells. Notably, H_2_O_2_-reduced ovarian size was reversed by PF. Our results are consistent with previous studies [33,34], which suggested that PF treatment can inhibit ovarian cell apoptosis and promote ovarian development. Furthermore, we found that PF increased PCNA expression levels in the ovary. PCNA is an important indicator for detecting cell proliferation status. Interestingly, PCNA was specifically localized in granulosa cells within follicles in this study, which may be related to the massive proliferation of granulosa cells during follicle development [35,36]. A previous study demonstrated that PF inhibits proliferation in breast cancer cells [37]. Wang et al. [38] also found that PF inhibits proliferation and promotes apoptosis in colon cancer cells, which is attributed to the anti-inflammatory effect of PF. However, we observed that PF promoted granulosa cell proliferation and follicle development in H_2_O_2_-induced ovarian oxidative damage. The results of this study are different from those of previous studies, which may be due to the previous studies focusing on cancer cells or mouse models [37,38]. These findings indicate that the protective effects and mechanisms of PF vary in different cells or organs. Previous studies generally believe that apoptosis is the main factor of follicular atresia [39,40]. Further results showed that there was no significant difference in the amount of follicle atresia before and after PF treatment (*p* > 0.05), indicating that PF can accelerate follicle development and inhibit ovarian cell apoptosis in the ovary, but it does not participate in regulating the process of follicular atresia.

Oxidative stress damages mitochondria, causing mitochondrial dysfunction. Cao et al. [41] confirmed that PF reduces ROS levels and increases mitochondrial membrane potential to restore the mitochondrial quality in macrophages. In this study, we observed consistent results with Cao et al. [41] that H_2_O_2_-induced oxidative stress caused mitochondrial dysfunction, while PF repaired H_2_O_2_-damaged mitochondria. Delayed ovarian development is accompanied by impaired autophagy. It is well known that autophagy plays a critical role in clearing damaged mitochondria. In recent years, many studies have confirmed that PF is involved in the regulation of autophagy activity [41,42,43]. LC3-II is used to analyze autophagy activity and is localized on the autophagosome membrane [44]. Beclin-1 participates in the extension of autophagosomes. p62, an autophagy protein substrate, was used to monitor autophagic flux [45]. The present study showed that PF upregulated LC3-II and Beclin-1 expression levels and reduced p62 protein accumulation, activating autophagic flux in ovarian cells. And PF increased TFEB and LAMP2 expression levels, suggesting that lysosomal biogenesis was increased, and autophagic turnover was normal. Our findings are consistent with those of Zhou et al. [46] that PF enhances autophagy activity to prevent adverse stimuli and promote cell survival. Sun et al. [47] reported that PF increases LC3 expression, enhances Parkin-mediated mitophagy, and rescues mitochondrial damage in PC12 cells. Our study found that PF upregulated mitophagy marker PINK1 and Parkin expression levels and increased the co-localization of LC3 and COX IV, which is consistent with Sun et al. [47]. A large number of studies have demonstrated that autophagy can prevent oxidative stress and improve cell function [48,49,50]. These findings indicate that PF-activated mitophagy clears damaged mitochondria and improves mitochondrial quality, thereby improving ovarian cell function. In the H_2_O_2_-induced ovarian oxidative stress model, oxidative damage is accompanied by impaired mitophagy, which further aggravates ovarian dysfunction, whereas PF activates mitophagy to resist oxidative stress and thereby promotes ovarian development.

## 4. Materials and Methods

### 4.1. Ethics Statement

All procedures involving animals were approved and conducted by the Institutional Animal Care and Use Committee of Zhejiang A&F University, China (ZAFUAC2023005).

### 4.2. Animals

Thirty female ICR mice (3 weeks old) were kept at 22 ± 2 °C under 12 h light/dark cycles. The mice received food and water ad libitum. All mice were weighed and randomly divided into 3 groups (n = 10): control, H_2_O_2_ treatment, and PF+ H_2_O_2_ treatment groups. H_2_O_2_ was given daily by intraperitoneal injection (i.p.) (48 mg/kg body weight) in the H_2_O_2_ and PF+ H_2_O_2_ groups. PF (CAS 23180-57-6, purity ≥ 98%, extracted from *Paeonia lactiflora* Pall., Shanghai yuanye Bio-Technology Co., Ltd., Shanghai, China) was then administered i.p. (10 mg/kg body weight) daily in the PF + H_2_O_2_ group. The same volume of normal saline was injected into the control group. After 7 weeks of treatment, the mice were weighed and euthanized. Ovarian tissues were collected and immediately weighed. The tissues were divided into two parts: (1) the tissues of part one were fixed in Bouin’s solution for histological assays; (2) the tissues of part two were frozen for Western blot.

### 4.3. Histology and Follicle Count

The fixed ovarian tissues were embedded and sliced (5 μm). Hematoxylin and eosin staining or histological observation was performed as described previously [51]. The images were captured with a microscope (CKX53SF, OLYMPUS, Tokyo, Japan). Serial sections of the ovaries were performed to count the number of follicles. The number of follicles at each stage was observed and counted under a microscope. Primordial follicles: a layer of flattened granulosa cells surrounding the oocyte. Primary follicles: the flat granulosa cells surrounding the oocyte become columnar granulosa cells. Secondary follicles: one layer of granulosa cells surrounding the oocyte becomes two to three layers; no follicular cavity. Antral follicles: multiple layers of granulosa cells surrounding the oocyte; clear follicular cavity.

### 4.4. TUNEL Assay

The apoptosis of ovarian cells was evaluated by TUNEL staining according to the manufacturer’s instructions. 4’,6-diamidino-2-phenylindole (DAPI, 1 μg/mL) was used to label the nuclear factor. The images were captured with a fluorescence microscope (CKX53SF, OLYMPUS, Tokyo, Japan). Six sections were randomly selected to detect cell apoptosis.

### 4.5. Immunofluorescence (IF)

For IF analysis, paraffin sections were deparaffinized and rehydrated. The nonspecific binding sites of sections were blocked with 5% bovine serum albumin for 30 min at 37 °C. The sections were incubated with primary antibody (Table 1) overnight at 4 °C, and then with secondary antibody IgG Cy3/FITC (1:500, bs-0296G-Cy3/FITC, BIOSS, Beijing, China) or IgG Cy3/FITC (1:500, bs-0295G-Cy3/FITC, BIOSS, Beijing, China). DAPI (1 μg/mL) was used to label the nuclear factor. The images were captured with a fluorescence microscope (CKX53SF, OLYMPUS, Tokyo, Japan).

### 4.6. Western Blot

The ovarian tissues were collected and lysed in RIPA (P0013B, Beyotime, Shanghai, China) containing PMSF (ST507, Beyotime, Shanghai, China). The lysates were centrifuged at 12,000× *g* at 4 °C. Then, 20 μg of total proteins was loaded and separated by 10-12 % SDS-PAGE. The gels were transferred to Nitrocellulose membranes (Millipore, Bedford, MA, USA). The blots were blocked with 5% nonfat milk for 1 h at room temperature. The membranes were incubated with primary antibody (Table 1) overnight at 4 °C, respectively. Subsequently, the membranes were incubated with secondary antibodies (1:10,000, CW0102S and CW0103S, CWBIO, Beijing, China) for 1 h at 37 °C. Immunoreactivity was detected by ECL (RM00020, Abclonal, Wuhan, China). Gray value quantization was performed using Image J 1.48v software.

### 4.7. Statistical Analysis

The data are presented as mean ± standard error of the mean (SEM). Statistical tests were performed using GraphPad Prism software (version 6). Differences were determined using a one-way analysis of variance (ANOVA). *p* < 0.05 was considered statistically significant. The experiments were repeated three times.

## 5. Conclusions

In summary, we confirmed that PF plays a protective effect in the ovarian development of the H_2_O_2_-induced mouse oxidative stress model. PF prevented oxidative stress, promoted granulosa cell proliferation, and thereby promoted follicle development. Furthermore, PF improved mitochondrial quality by activating mitophagy activity. Thus, our study provides new evidence that PF promotes ovarian development, and provides a theoretical basis for improving female reproductive capacity.

## Figures and Tables

**Table 1:** Antibodies used in this study.

For Immunofluorescence Assay			
Antibody	Dilution	Cat No.	Company
PCNA	1:500	GB11010	Servicebio, Wuhan, China
HO-1	1:400	GB12104	Servicebio, Wuhan, China
Nrf2	1:300	16396-1-AP	Proteintech, Rosemont, IL, USA
Beclin-1	1:300	66665-1-Ig	Proteintech, Rosemont, IL, USA
p62	1:500	18420-1-AP	Proteintech, Rosemont, IL, USA
LAMP2	1:400	GB11330	Servicebio, Wuhan, China
PINK1	1:300	GB114934	Servicebio, Wuhan, China
Parkin	1:200	66674-1-Ig	Proteintech, Rosemont, IL, USA
LC3	1:100	A19665	Abclonal, Wuhan, China
COX IV	1:500	GB12250	Servicebio, Wuhan, China
γH2A	1:500	GB111841	Servicebio, Wuhan, China
**For Western blot assay**			
TOM20	1:3000	A19403	Abclonal, Wuhan, China
MFN1	1:4500	13798-1-AP	Proteintech, Rosemont, IL, USA
DRP1	1:1000	8570S	Cell Signaling Technology, Danvers, MA, USA
TFEB	1:1000	ER65144	HUABIO, Hangzhou, China
p62	1:10,000	18420-1-AP	Proteintech, Rosemont, IL, USA
LC3	1:1000	A19665	Abclonal, Wuhan, China
GPX4	1:1000	30388-1-AP	Proteintech, Rosemont, IL, USA
BCL2	1:4000	12789-1-AP	Proteintech, Rosemont, IL, USA
BAX	1:6000	50599-2-Ig	Proteintech, Rosemont, IL, USA
ACTB	1:5000	20536-1-AP	Proteintech, Rosemont, IL, USA

## Data Availability

All data generated or analyzed during this study are available from the corresponding author on request.

## Abbreviations

ACTB: β-actin; Akt: Protein kinase B; BCL2: B-cell lymphoma 2; BAX: BCL2-Associated X; COX IV: Cytochrome c oxidase polypeptide IV; DRP1: Dynamin-related protein 1; GPX4: Glutathione Peroxidase 4; HO-1: heme oxygenase-1; ROS: reactive oxygen species; SDS-PAGE: Sodium dodecyl-sulfate polyacrylamide gel electrophoresis; SEM: standard error of the mean; Smad3: SMAD family member 3; TGF-β1: transforming growth factor-beta 1; TOM20: Translocase of outer mitochondrial membrane 20; TFEB: transcription factor EB; LAMP2: Lysosome-associated membrane protein 2; LC3: Microtubule-associated protein 1A/1B-light chain 3; MFN1: Mitofusin 1; Nrf2: nuclear factor erythroid 2-related factor 2; PCNA: Proliferating cell nuclear antigen; p62: SQSTM1, sequestosome 1; PINK1: PTEN-induced kinase 1; PF: Paeoniflorin; γH2A: Phosphorylation of histone H2A.

## References

[1] Wang L., Tang J., Wang L., Tan F., Song H., Zhou J., Li F. (2021). Oxidative stress in oocyte aging and female reproduction. J. Cell Physiol..

[2] Dutta S., Sengupta P., Slama P., Roychoudhury S. (2021). Oxidative Stress, Testicular Inflammatory Pathways, and Male Reproduction. Int. J. Mol. Sci..

[3] Mailloux R.J. (2020). An Update on Mitochondrial Reactive Oxygen Species Production. Antioxidants.

[4] Tao Y., Pan Y., Wang Q., Lu S., Li Y., Liu W., Zheng T., Wang B., Qiang J., Xu P. (2023). Vitamin E Ameliorates Impaired Ovarian Development, Oxidative Stress, and Disrupted Lipid Metabolism in Oreochromis niloticus Fed with a Diet Containing Olive Oil Instead of Fish Oil. Antioxidants.

[5] Silva B.R., Silva J.R.V. (2023). Mechanisms of action of non-enzymatic antioxidants to control oxidative stress during in vitro follicle growth, oocyte maturation, and embryo development. Anim. Reprod. Sci..

[6] Barrozo L.G., Paulino L.R., Silva B.R., Barbalho E.C., Nascimento D.R., Neto M.F.L., Silva J.R. (2021). N-acetyl-cysteine and the control of oxidative stress during in vitro ovarian follicle growth, oocyte maturation, embryo development and cryopreservation. Anim. Reprod. Sci..

[7] Hayashi S., Nakamura T., Motooka Y., Ito F., Jiang L., Akatsuka S., Iwase A., Kajiyama H., Kikkawa F., Toyokuni S. (2020). Novel ovarian endometriosis model causes infertility via iron-mediated oxidative stress in mice. Redox Biol..

[8] Devine P.J., Perreault S.D., Luderer U. (2012). Roles of reactive oxygen species and antioxidants in ovarian toxicity. Biol. Reprod..

[9] Shen M., Lin F., Zhang J., Tang Y., Chen W.K., Liu H. (2012). Involvement of the up-regulated FoxO1 expression in follicular granulosa cell apoptosis induced by oxidative stress. J. Biol. Chem..

[10] Jiang Y., Shen M., Chen Y., Wei Y., Tao J., Liu H. (2021). Melatonin Represses Mitophagy to Protect Mouse Granulosa Cells from Oxidative Damage. Biomolecules.

[11] Hou L., Gu T., Weng K., Zhang Y., Zhang Y., Chen G., Xu Q. (2023). Effects of Oxidative Stress on the Autophagy and Apoptosis of Granulosa Cells in Broody Geese. Int. J. Mol. Sci..

[12] Zhang J.-Q., Shen M., Zhu C.-C., Yu F.-X., Liu Z.-Q., Ally N., Sun S.-C., Li K., Liu H.-L. (2014). 3-Nitropropionic acid induces ovarian oxidative stress and impairs follicle in mouse. PLoS ONE.

[13] Choi J., Jo M., Lee E., Choi D. (2014). AKT is involved in granulosa cell autophagy regulation via mTOR signaling during rat follicular development and atresia. Reproduction.

[14] Gawriluk T.R., Hale A.N., Flaws J.A., Dillon C.P., Green D.R., Rucker E.B. (2011). Autophagy is a cell survival program for female germ cells in the murine ovary. Reproduction.

[15] Gioia L., Festuccia C., Colapietro A., Gloria A., Contri A., Valbonetti L. (2019). Abundances of autophagy-related protein LC3B in granulosa cells, cumulus cells, and oocytes during atresia of pig antral follicles. Anim. Reprod. Sci..

[16] Ren S., Wang Y., Zhang Y., Yan P., Xiao D., Zhao Y., Jia W., Ding L., Dong H., Wei C. (2023). Paeoniflorin alleviates AngII-induced cardiac hypertrophy in H9c2 cells by regulating oxidative stress and Nrf2 signaling pathway. Biomed. Pharmacother..

[17] Zhang L., Wei W. (2020). Anti-inflammatory and immunoregulatory effects of paeoniflorin and total glucosides of paeony. Pharmacol. Ther..

[18] Liu Z., Gao J., Ban Y., Wan T.T., Song W., Zhao W., Teng Y. (2024). Synergistic effect of paeoniflorin combined with luteolin in alleviating Lipopolysaccharides-induced acute lung injury. J. Ethnopharmacol..

[19] Jiang J., Dong C., Zhai L., Lou J., Jin J., Cheng S., Chen Z., Guo X., Lin D., Ding J. (2021). Paeoniflorin Suppresses TBHP-Induced Oxidative Stress and Apoptosis in Human Umbilical Vein Endothelial Cells via the Nrf2/HO-1 Signaling Pathway and Improves Skin Flap Survival. Front. Pharmacol..

[20] Wang L., An H., Yu F., Yang J., Ding H., Bao Y., Xie H., Huang D. (2022). The neuroprotective effects of paeoniflorin against MPP(+)-induced damage to dopaminergic neurons via the Akt/Nrf2/GPX4 pathway. J. Chem. Neuroanat..

[21] Zhou J., Tan Y., Wang X., Zhu M. (2021). Paeoniflorin attenuates DHEA-induced polycystic ovary syndrome via inactivation of TGF-β1/Smads signaling pathway in vivo. Aging.

[22] Wu Q., Chen M., Li Y., Zhao X., Fan C., Dai Y. (2023). Paeoniflorin Alleviates Cisplatin-Induced Diminished Ovarian Reserve by Restoring the Function of Ovarian Granulosa Cells via Activating FSHR/cAMP/PKA/CREB Signaling Pathway. Molecules.

[23] Park M.J., Han S.-E., Kim H.J., Heo J.D., Choi H.-J., Ha K.-T., Yang S.W., Lee K.S., Kim S.C., Kim C.W. (2020). Paeonia lactiflora improves ovarian function and oocyte quality in aged female mice. Anim. Reprod..

[24] Wang X., Yang J., Li H., Mu H., Zeng L., Cai S., Su P., Li H., Zhang L., Xiang W. (2023). miR-484 mediates oxidative stress-induced ovarian dysfunction and promotes granulosa cell apoptosis via SESN2 downregulation. Redox Biol..

[25] Zhang S., Liu Q., Chang M., Pan Y., Yahaya B.H., Liu Y., Lin J. (2023). Chemotherapy impairs ovarian function through excessive ROS-induced ferroptosis. Cell Death Dis..

[26] Niu C., Jiang D., Guo Y., Wang Z., Sun Q., Wang X., Ling W., An X., Ji C., Li S. (2023). Spermidine suppresses oxidative stress and ferroptosis by Nrf2/HO-1/GPX4 and Akt/FHC/ACSL4 pathway to alleviate ovarian damage. Life Sci..

[27] Xu B., He T., Yang H., Dai W., Liu L., Ma X., Ma J., Yang G., Si R., Du X. (2023). Activation of the p62-Keap1-Nrf2 pathway protects against oxidative stress and excessive autophagy in ovarian granulosa cells to attenuate DEHP-induced ovarian impairment in mice. Ecotoxicol. Environ. Saf..

[28] Chen X., Song Q.L., Li Z.H., Ji R., Wang J.Y., Cao M.L., Mu X.F., Zhang Y., Guo D.Y., Yang J. (2023). Pterostilbene ameliorates oxidative damage and ferroptosis in human ovarian granulosa cells by regulating the Nrf2/HO-1 pathway. Arch. Biochem. Biophys..

[29] Wang Y., Yan S., Liu X., Deng F., Wang P., Yang L., Hu L., Huang K., He J. (2022). PRMT4 promotes ferroptosis to aggravate doxorubicin-induced cardiomyopathy via inhibition of the Nrf2/GPX4 pathway. Cell Death Differ..

[30] Ma L., Liu X., Zhang M., Zhou L., Jiang L., Gao L., Wang X., Huang Y., Zeng H., Wu Y. (2023). Paeoniflorin alleviates ischemia/reperfusion induced acute kidney injury by inhibiting Slc7a11-mediated ferroptosis. Int. Immunopharmacol..

[31] Zeng Q., Hong W. (2008). The emerging role of the hippo pathway in cell contact inhibition, organ size control, and cancer development in mammals. Cancer Cell..

[32] Hussein M.R. (2005). Apoptosis in the ovary: Molecular mechanisms. Hum. Reprod. Update.

[33] Zhou H.-Q., Liu W., Wang J., Huang Y.-Q., Li P.-Y., Zhu Y., Wang J.-B., Ma X., Li R.-S., Wei S.-Z. (2017). Paeoniflorin attenuates ANIT-induced cholestasis by inhibiting apoptosis in vivo via mitochondria-dependent pathway. Biomed. Pharmacother..

[34] Hua X., Feng X., Hua Y., Wang D. (2023). Paeoniflorin attenuates polystyrene nanoparticle-induced reduction in reproductive capacity and increase in germline apoptosis through suppressing DNA damage checkpoints in Caenorhabditis elegans. Sci. Total Environ..

[35] Zhang C.-P., Yang J.-L., Zhang J., Li L., Huang L., Ji S.-Y., Hu Z.-Y., Gao F., Liu Y.-X. (2011). Notch signaling is involved in ovarian follicle development by regulating granulosa cell proliferation. Endocrinology.

[36] Lv X., He C., Huang C., Wang H., Hua G., Wang Z., Zhou J., Chen X., Ma B., Timm B.K. (2019). Timely expression and activation of YAP1 in granulosa cells is essential for ovarian follicle development. FASEB J..

[37] Zhang Q., Yuan Y., Cui J., Xiao T., Jiang D. (2016). Paeoniflorin inhibits proliferation and invasion of breast cancer cells through suppressing Notch-1 signaling pathway. Biomed. Pharmacother..

[38] Wang Y., Zhou Y., Lin H., Chen H., Wang S. (2022). Paeoniflorin Inhibits the Proliferation and Metastasis of Ulcerative Colitis-Associated Colon Cancer by Targeting EGFL7. J. Oncol..

[39] Hułas-Stasiak M., Gawron A. (2011). Follicular atresia in the prepubertal spiny mouse (*Acomys cahirinus*) ovary. Apoptosis Int. J. Program. Cell Death.

[40] Inoue N., Matsuda F., Goto Y., Manabe N. (2011). Role of cell-death ligand-receptor system of granulosa cells in selective follicular atresia in porcine ovary. J. Reprod. Dev..

[41] Cao Y., Xiong J., Guan X., Yin S., Chen J., Yuan S., Liu H., Lin S., Zhou Y., Qiu J. (2023). Paeoniflorin suppresses kidney inflammation by regulating macrophage polarization via KLF4-mediated mitophagy. Phytomedicine.

[42] Wang X., Jiang L., Liu X.-Q., Huang Y.-B., Wang A.-L., Zeng H.-X., Gao L., Zhu Q.-J., Xia L.-L., Wu Y.-G. (2022). Paeoniflorin binds to VEGFR2 to restore autophagy and inhibit apoptosis for podocyte protection in diabetic kidney disease through PI3K-AKT signaling pathway. Phytomedicine.

[43] Zhao F., Peng C., Li H., Chen H., Yang Y., Ai Q., Chen N., Liu F. (2023). Paeoniae Radix Rubra extract attenuates cerebral ischemia injury by inhibiting ferroptosis and activating autophagy through the PI3K/Akt signalling pathway. J. Ethnopharmacol..

[44] Mizushima N., Yoshimori T. (2007). How to interpret LC3 immunoblotting. Autophagy.

[45] Jiang T., Harder B., Rojo de la Vega M., Wong P.K., Chapman E., Zhang D.D. (2015). p62 links autophagy and Nrf2 signaling. Free Radic. Biol. Med..

[46] Zhou X., Chen X., Cheng X., Lin L., Quan S., Li S., Zhan R., Wu Q., Liu S. (2023). Paeoniflorin, ferulic acid, and atractylenolide III improved LPS-induced neuroinflammation of BV2 microglia cells by enhancing autophagy. J. Pharmacol. Sci..

[47] Sun R., Liu J., Yu M., Xia M., Zhang Y., Sun X., Xu Y., Cui X. (2022). Paeoniflorin Ameliorates BiPN by Reducing IL6 Levels and Regulating PARKIN-Mediated Mitochondrial Autophagy. Drug Des. Dev. Ther..

[48] Albano G.D., Montalbano A.M., Gagliardo R., Profita M. (2023). Autophagy/Mitophagy in Airway Diseases: Impact of Oxidative Stress on Epithelial Cells. Biomolecules.

[49] Talebi M., Vadoud S.A.M., Haratian A., Talebi M., Farkhondeh T., Pourbagher-Shahri A.M., Samarghandian S. (2022). The interplay between oxidative stress and autophagy: Focus on the development of neurological diseases. Behav. Brain Funct. BBF.

[50] Peters A.E., Mihalas B.P., Bromfield E.G., Roman S.D., Nixon B., Sutherland J.M. (2020). Autophagy in Female Fertility: A Role in Oxidative Stress and Aging. Antioxid. Redox Signal..

[51] Yang Q.E., Gwost I., Oatley M.J., Oatley J.M. (2013). Retinoblastoma protein (RB1) controls fate determination in stem cells and progenitors of the mouse male germline. Biol. Reprod..

